# Visual Cues Promote Head First Strategies During Walking Turns in Individuals With Parkinson's Disease

**DOI:** 10.3389/fspor.2020.00022

**Published:** 2020-03-11

**Authors:** Tyler Baker, Jenna Pitman, Michael James MacLellan, Rebecca J. Reed-Jones

**Affiliations:** ^1^Department of Applied Human Sciences, Faculty of Science, University of Prince Edward Island, Charlottetown, PE, Canada; ^2^Department of Human Health and Nutritional Sciences, College of Biological Science, University of Guelph, Guelph, ON, Canada

**Keywords:** visual cues, walking turns, kinematics, Parkinsons' disease, motor control, locomotion ability

## Abstract

Anticipatory eye movement promotes cranio-caudal sequencing during walking turns. Clinical groups, such as Parkinson's disease (PD), do not produce anticipatory eye movements, leading to increased risk of falls. Visual cues may promote anticipatory eye movement by guiding the eyes into the turn. This study examined if visual cues could train anticipatory eye movement. Ten neurotypical young adults and 6 adults with PD completed three blocks of walking trials. Trials were blocked by visual condition: non-cued baseline turns (5 trials), visually cued turns (10 trials), and non-cued post turns (5 trials). A Delsys Trigno (Delsys, Boston, MA) recorded horizontal saccades at 1024 Hz via electrooculography (EOG). Two Optotrak cameras (Northern Digital Inc., ON, Canada) captured body segment kinematics at 120 Hz. Initiation of segment rotation with respect to ipsilateral foot contact (IFC1) prior to the turn was calculated. Neurotypical young adults (NYA) produced typical cranio-caudal rotation sequences during walking turns. Eyes led (407 ms prior to IFC1), followed by the head (50 ms prior to IFC1), then trunk and pelvis. In contrast, PD produced no anticipatory eye or segment movement at baseline. During pre-trials the eyes moved 96 ms after IFC1 and segment movement was initiated by the pelvis followed by trunk and head segments. After visual cue training however, PD produced anticipatory eye movements 161 ms prior to IFC1, followed by the head 88 ms following IFC1 but ahead of trunk and pelvis onset. These results suggest visual cues assist in producing cranio-caudal control during walking turns in PD.

## Introduction

Changing direction, or turning, while walking is a critical aspect of mobility. Turns can comprise up to 45 percent of the total steps taken in a given day (Glaister et al., 2007). To complete a turn while walking the central nervous system (CNS) must coordinate reorientation of the body's segments in the new direction of travel, while at the same time maintaining postural control and the stepping sequence (Patla et al., 1999; Mellone et al., 2016). The most common turning angle used during activities of daily living is a 90-degree turn which is also considered the most unstable given the rapid change in direction that can lead to lateral falls (Mellone et al., 2016).

Turning while walking is defined as having a robust cranio-caudal segment sequence resulting in a head first turning strategy (Spildooren et al., 2017) or steering synergy (Patla et al., 1999; Ambati et al., 2016). This strategy requires independent control of the segments, increasing the complexity of control by the CNS, but also allows individuals to respond to perturbations (Assaiante and Amblard, 1995). It is therefore no surprise that turning while walking becomes difficult through the natural aging of the CNS as well as for individuals with CNS deficits and/or diseases. For example, individuals with Parkinson's disease present an “en-bloc” turning strategy defined as the ascending organization of segment reorientation where the head and body reorient at the same time (or as one) in a blocked or single unit fashion (Assaiante and Amblard, 1995; Spildooren et al., 2017). The use of this strategy serves to minimize the degrees of freedom the CNS must control and aids in postural control. However, in real life situations where adjustments in movement are made quickly, an “en-bloc” strategy may be unsafe and lead to more falls (Assaiante and Amblard, 1995; Wright et al., 2012; Akram et al., 2014).

### The Role of Vision in Turning While Walking

During walking and turning, gaze shifts, and reorientation of the head to the new direction of travel prior to turning the body provides the CNS with a frame of reference to assist reorientation of the rest of the body (Hollands et al., 2002). Neurotypical young adults can initiate gaze redirection up to 1000 ms prior to turning (Grasso et al., 1998; Reed-Jones et al., 2009). However, individuals with Parkinson's disease, unlike neurotypical young adults, do not anticipate a turn by moving their eyes and head in the direction of the turn. Instead, individuals with PD maintain the eyes straight ahead until the onset of the turn and use an “en-bloc” turning strategy (Ambati et al., 2016). These observations suggest that en-bloc segment coordination may result from a lack of anticipatory redirection of the eyes. Therefore, developing visual cueing strategies to specifically target anticipatory eye movement while turning may promote use of a head first turning strategy thereby reducing fall risk.

The purpose of this study was to explore how discrete external visual cues would influence acute turning abilities in a group with PD. It was anticipated that the use of discrete external visual cueing would promote eye movement and result in a cranio-caudal rotation sequence deterring PD from using an “en-bloc” movement strategy. In addition to PD, a neurotypical young adult (NYA) group was also examined to understand how the use of visual cues may influence individuals who already use robust cranio-caudal sequencing during walking turns.

## Materials and Methods

### Participants

This study consisted of two sample groups. A neurotypical young adult (NYA) group consisting of 5 male and 5 female volunteers (22.4 +/– 1.4 years), and a group of individuals affected by Parkinson's disease (PD) consisting of 3 male and 3 female (60.8 +/– 11.7 years) whose H&Y stages ranged from 1-3 (Bhidayasiri and Tarsy, 2012) as assessed by the Unified Parkinson's Disease Rating Scale (UPDRS) questionnaire (Goetz et al., 1995). Prior to the study, all participants were screened to identify any medical conditions which would put them at risk by participating in the study. If a risk factor was detected, the participant was excluded for safety reasons. Approval was obtained from the University of Prince Edward Island Research Ethics Board and all participants provided informed written consent prior to data collection. Data collection was conducted in accordance with the World Medical Association's Declaration of Helsinki.

### Experimental Set-Up

A course consisting of a straight walkway leading to a 90-degree left turn was constructed. The dimensions of the straight walkway were 0.95 meters in width and 2.29 meters in length. The walkway after the turn was 1.37 meters in length (See Supplemental Data for schematic and pictures). Participants were instructed to proceed down the walkway at an unconstrained self-selected pace until they reached the 90-degree left turn.

Four discrete external visual cues (large high contrast numbers 1, 2, 3, 4), printed on 11 × 17 inch paper and laminated, were placed on the laboratory wall with a barrier being used to obstruct three of the four cues (2, 3, 4) from the participants' view. Cue 2 became visible to the participant just before they entered the turn while cue 3 and 4 were visible once the participant proceeded into the turn. Hiding the visual cues was done to promote anticipatory eye movements during turning and to eliminate visual searching and/or sampling at the beginning of the trials.

Two Optotrak (Northern Digital Inc., Waterloo, ON) cameras captured three-dimensional kinematic data at 120 Hz. Five triangular rigid bodies with three active Infrared Emitting Diode (IRED) markers were secured to the anterior aspect of the head, trunk, pelvis and right, and left foot of the participant. A further marker was placed on each heel for heel contact event detection. A Delsys Trigno (Delsys, Boston, MA) Wireless Electrocardiography (ECG) sensor was used to record electrooculography (EOG) at a frequency of 1024 Hz. The electrode was placed on the lateral aspect of the orbit to track horizontal movement of the eye. Calibration of EOG was done with a 9-point calibration reference frame typically used for eye tracking devices so that forward gaze produced steady baseline data, a shift of the eyes to the right produced positive values and a shift of the eyes to the left produced negative values.

### Protocol

Participants were instructed to proceed to the starting line of the course in preparation for each trial. The researcher counted down from three, at which point the participant walked toward the turn. At the turning point, they completed a left turn around the 90-degree angle.

Participants were permitted up to three practice trials before data collection began. These practice trials were used to determine the start foot (first step in trial) so that the left foot would fall at the point in the walkway for the participant to begin the turn.

Walking trials consisted of three sets of blocked trials: Five pre trials where participants walked and turned on the walkway in their own manner (“pre”), followed by ten trials with visual cues in place (“cued”), and finally five post trials where visual cues had been removed (“post”).

On completion of the first 5 pre trials, participants were asked to stand or sit near the starting line of the course for a short 5-min break. During this break, the visual cues (1, 2, 3, 4) were placed in their appropriate locations. After this break, the participant was asked to stand at the start position and instructed to “look at the cues as they became visible”. During this set of trials, as the participant walked down the course toward the turn, they saw a visual cue on the wall in front of them with the number 1 on it. As they approached the turn, proceeded into the turn, and completed the left turn, cues 2, 3, and 4 located on the wall, became visible to the participant. This protocol was carried out for the ten cued trials. Following completion of the cued trials each participant was allotted a mandatory 15-min break in the testing which allowed for a brief washout period. During this break, the neurotypical young adult participants sat quietly, whereas, all participants with PD sat and completed the Activities-Specific Balance Confidence (ABC) Scale (Powell and Myers, 1995). All PD reported high levels of confidence (70–100%) and the ABC scale was not used in further data analyses. Once the 15-min break was completed, participants proceeded back to the starting line of the course for the final set of trials. The same protocol was used as those of the initial pre trials.

### Data Reduction

The turn movement was defined by the ipsilateral foot contact (IFC1) prior to change in direction (onset) to the ipsilateral foot contact (IFC2) following the change in direction (completion), as used in previous work (Patla et al., 1999; Ambati et al., 2016). Refer to Supplementary Figure 1 in supplementary files for further definition. Foot contacts were determined as the zero crossing following minima of the heel marker vertical velocity.

All kinematic data were exported from First Principles (Northern Digital Inc., Waterloo, ON) collection software and imported into a custom Matlab program. Kinematic data were smoothed using a second-order, dual-pass Butterworth filter with a low-pass cut off of 7 Hz. Yaw angular displacement (about the global vertical axis) were calculated for the head, trunk and pelvis using the triad markers placed on each segment. These were numerically differentiated to determine angular velocities at each instant in time. Time of turn onset was then estimated as the local minima of each segment's yaw angular velocity prior to reaching maximum angular velocity during the turning motion. Segment onsets were referenced with respect to IFC1 by subtracting each segment onset time from IFC1 time (milliseconds) as per previously published work (Patla et al., 1999; Hollands et al., 2002; Ambati et al., 2016). Intersegment timing, differences between when adjacent segments initiated rotation, were examined by comparing the onset of the inferior segment to that of the superior segment. In addition to timing variables, magnitude of segment rotation, and turn time (duration of the turning movement) were calculated. Turn magnitude was the final angular displacement of the segments at IFC2 (completion of turn). Turn time was calculated by subtracting time at IFC2 from IFC1.

**Figure 1:**
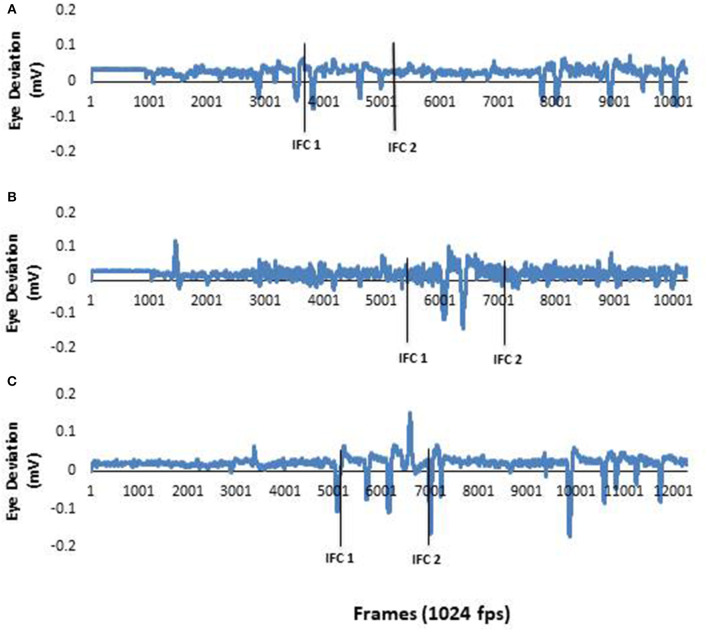
**(A)** EOG data from a typical neurotypical young adult participant. As the healthy young adult participants eye movements were active throughout the study, these data represent information collected in all three conditions. **(B)** EOG data from a typical PD participant during a pre-trial. There was no horizontal eye movement in the first half of the recording. This demonstrated there was no anticipatory eye movements used by the PD during this trial. **(C)** EOG data from a PD participant in **(B)** during a post trial. This figure clearly shows anticipatory eye movement during the trial. The first solid black line represents ipsilateral foot contact I (IFCI) and the second solid black line represents ipsilateral foot contact 2 (IFC2). The two lines together window the 90 degree left tum.

Analysis of EOG focused on horizontal saccadic eye movement. Saccade onset was determined from the zero-crossing of the first minima in the EOG data. EOG data was manually inspected by a single rater. This onset time was then calculated with respect to IFC1 as per the body segments. Representative raw eye data are presented in Figure 1. The raw data supporting the conclusions of this manuscript will be made available by the authors, without undue reservation, to any qualified researcher.

### Statistical Analyses

All participants reached a maximum segment rotation magnitude of 1.6 rads (91.7 degrees) and fully completed the 90 degree turn. Rotation magnitudes were not analyzed further.

A general linear model repeated measures analysis of variance (ANOVA) was used to examine the effects of visual cues *within* each of the participant groups: the neurotypical young adult group and the group with Parkinson's disease. This model was used because the interest of this study was not to compare between these two groups, but rather to understand the effects of the visual cues within each group. For each group the independent variable *visual cues* had 3 levels: pre, cued, and post. The dependent variables - onset times of the eyes, head, trunk, and pelvis were assessed using a univariate model. Alpha level was set at 0.05 and Greenhouse-Geisser corrections were used when assumptions of sphericity were violated. Pair-wise comparisons were made using Bonferroni corrections for multiple comparisons. For all statistical reporting the effect size is included (*n*^*2*^).

To address the question of whether a top-down segment sequence was used we compared the onset time of the inferior segment to the onset time of the superior segment using paired sample t-tests. For example, a paired sample *t*-tests between the eyes and head were completed for each of the conditions pre, cued, and post. Non-significant differences between adjacent segment onset times suggest segments moving closer together (more en bloc) whereas significant differences suggest segments moving more independently.

To investigate the role condition played in the time required for NYA and PD to complete the 90-degree left turn, a general linear model repeated measures ANOVA was used with the independent variable visual condition set at 3 levels: pre, cued, and post and the dependent variable of time to complete the turn.

## Results

In order to address our research questions, data reporting is presented in a two-step manner. First, we describe the changes that occurred in the neurotypical young adult group as the group served as our baseline. Next, we describe the PD sample to determine how the visual cues altered their turning control.

### Comparison Within the Neurotypical Young Adult Sample

Raw eye data from a representative NYA (Figure 1) illustrates the distinct saccades made by NYA in the pre-trials. These distinct saccades were observed for all trials in NYA. Examination of eye and body segment rotation onset was done through repeated measures analysis with all dependent variables entered into the model. Analysis of segment onset time revealed no significant differences for EOG [*F*_(1.808, 16.276)_ = 1.800; *p* = 0.198; *n*^*2*^ = 0.167]; Head [*F*_(1.894, 17.044)_ = 1.195; *p* = 0.324; *n*^*2*^ = 0.117]; Trunk [*F*_(1.598, 14.386)_ = 1.090; *p* = 0.348; *n*^*2*^ = 0.108]; Pelvis [*F*_(1.812, 16.307)_ = 2.551; *p* = 0.113; *n*^*2*^ = 0.221].

**Figure 2:**
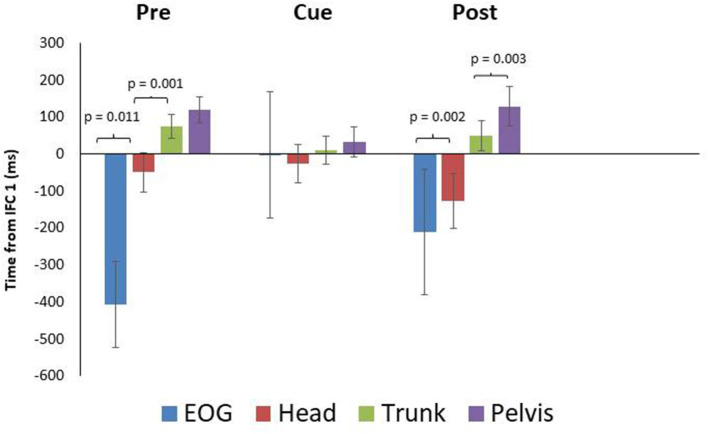
Eye and segment rotation onset (mean ± SE) with respect to IFC 1 of the walking turn for the neurotypical young adult (NYA) participants. Negative values indicate onset prior to initiation of the turn while positive values indicate onset after the start of the turn (IFC 1) as defined in this study. NYA produced a cranio-caudal rotation sequence throughout the trials. Anticipatory eye movements were delayed during trials with visual cues.

While onset times were not significantly different between conditions, there were some differences in timing worth noting as a result of visual cueing. Mean onset time (ms) for EOG during the pre, cued, and post trials were −407 ± 116 SEM, −3 ± 171 SEM, and −210 ± 169 SEM prior to IFC1 respectively (Figure 2), indicating that in the cued trials it took longer for the initial eye movement to occur. Mean onset time (ms) for head rotation were −50 ± 53 SEM, −27 ± 52 SEM, and −127 ± 75 SEM for the pre, cued, and post trials. These results indicated that NYA participants rotated their heads prior to the 90-degree left turn in all conditions; however, the earliest rotation was in the post trials (Figure 2). In all conditions, the eyes and head always led the body during turning, demonstrating robust use of the head first or steering synergy as defined by the literature (Grasso et al., 1998; Patla et al., 1999; Hollands et al., 2002; Reed-Jones et al., 2009).

Paired samples *T*-tests for intersegment coordination indicated there was a significant difference (*p* = 0.011) between EOG and head in the pre-trials but not in the cued (*p* = 0.889) or post trials (*p* = 0.637) (Figure 2). The head and trunk produced significant differences for the pre and post trials, but not for the cued trials (pre: *p* = 0.001, cue: *p* = 0.210, post: *p* = 0.002). The trunk and pelvis showed non-significant differences for the pre and cued trials, whereas a significant difference was found for post trials (pre: *p* = 0.082, cue: *p* = 0.134, post: *p* = 0.012).

**Figure 3:**
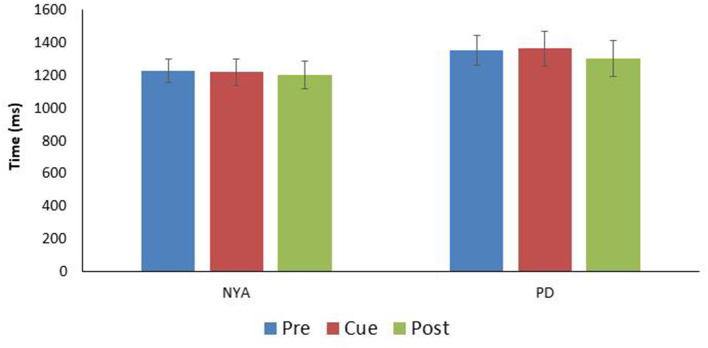
Comparison turn time (time between IFC1and IFC 2) for the two experimental groups (mean ± SE). Note there were no significant differences in turn time between the conditions within either group.

Finally, the time to complete the turn was examined between conditions (Figure 3). Results indicated that condition did not affect the time needed to complete the 90-degree walking left turn [F_(1.905, 17.147)_ = 0.496; *p* = 0.609; *n*^*2*^ = 0.052].

### Comparison Within the Parkinsonian Sample

Raw eye data from a representative PD (Figure 1) illustrates the absence of distinct saccades in the pre-trials. In contrast distinct saccades were observed in post trials. Repeated measures analysis indicated no significant effects of visual condition on EOG [*F*_(1.260, 6.302)_ = 0.927; *p* = 0.396; *n*^2^ = 0.156]; Head [*F*_(1.033, 5.165)_ = 1.521; *p* = 0.272; *n*^2^ = 0.233]; Trunk [*F*_(1.936, 9.681)_ = 1.391; *p* = 0.293; *n*^2^ = 0.218]; Pelvis [*F*_(1.279, 6.395)_ = 2.092; *p* = 0.199; *n*^2^ = 0.295].

**Figure 4:**
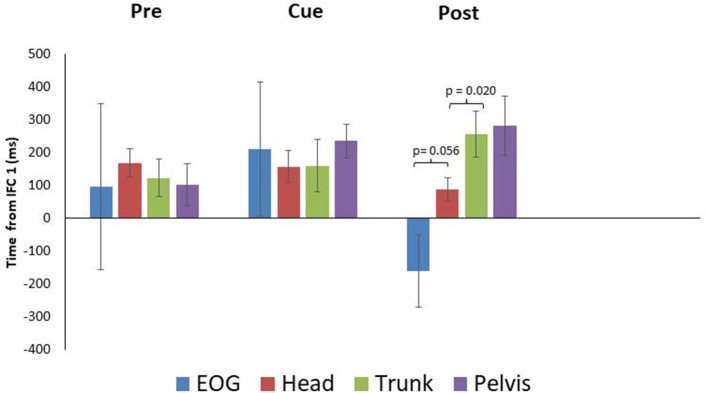
Eye and segment rotation onsets (mean ± SE) with respect to IFC 1of the walking turn for the Parkinsonian {PD} participants. Negative values indicate onset prior to initiation of the turn while positive values indicate onset prior to the start of the turn as defined in this study. PD produced a bottom up rotation sequence during pre-trials. However, following walking turns with visual cues (Cue), anticipatory eye movements and a cranio-caudal rotation sequence were observed (Post).

PD means for EOG were 96 ± 253 SEM, 210 ± 203 SEM, and−161 ± 109 SEM ms for the pre, cued and post trials, respectively. These data indicate PD did not produce anticipatory eye movement in the pre and cued trials as onset times followed IFC1. For post however, PD did produce anticipatory eye movement (Figures 1, 4). Head rotation initiated at 168 ± 43 SEM, 157 ± 49 SEM, and 88 ± 36 SEM ms following IFC 1 for each condition (pre, cued, and post) respectively (Figure 4). These data indicate that PD began to rotate their heads earlier with visual cues, with the earliest head rotation occurring in post trials. Trunk and pelvis onsets got progressively later as eye and head onsets got earlier from pre to cued to post trials (Figure 4). These results indicate PD began turning (in pre trials) with the pelvis and trunk moving first supporting more of an ascending bottom up turning control but finished trials (after visual cue training) with better top-down head first turning control.

Paired samples T-tests indicated a non-significant difference between EOG and head for the pre and cued trials, with marginal significance in post trials (pre: *p* = 0.753, cue: *p* = 0.762, post: *p* = 0.056) (Figure 4). The head and trunk produced a non-significant difference for the pre and cued trials, however, a significant difference was observed for the post trials (pre: *p* = 0.124, cue: *p* = 0.956, post: *p* = 0.020). The trunk and pelvis showed non-significant differences for all conditions (pre: *p* = 0.137, cue: *p* = 0.164, post: *p* = 0.538). The estimated marginal means indicted that the head rotated prior to the eyes in the cued (−53 ms) trials, however in the pre (72 ms), and post (249 ms) trials, the head rotated subsequent to the eyes.

Finally, the time to complete the turn was examined between conditions (Figure 3). Results indicated that condition did not affect the time needed to complete the 90-degree walking left turn [*F*_(1.905, 17.147)_ = 0.496; *p* = 0.609; n^2^ = 0.052].

## Discussion

The purpose of this study was to explore the use of discrete external visual cues to promote eye movements during a walking turn in a group with PD, thus reinforcing a head first strategy (Ambati et al., 2016; Spildooren et al., 2017). Neurotypical young adults were examined as a basis for understanding how visual cues may influence robust turning control. Interestingly the results suggest that the greatest effect of using visual cues during walking turns in a PD group was in trials following their use when the cues were removed (i.e., a post training effect).

### Neurotypical Young Adults

Neurotypical young adults naturally produce a head first strategy (Patla et al., 1999; Hollands et al., 2002; Ambati et al., 2016; Spildooren et al., 2017). Given this, we wanted to see how NYA would react to discrete external visual cues during walking turns. It was hypothesized NYA would not significantly alter turning behaviors between cued and non-cued trials. Results support this hypothesis partially. There were no significant main effects of condition on the onset of segment rotation with reference to IFC1. However, NYA did show significant differences in intersegment onset times. NYA produced anticipatory eye movement prior to head rotation onset as per the head first turning strategy in both pre and post conditions (Figure 2). However, there were some changes in the behavior of the eyes and head when visual cues were present that warrant discussing for future studies.

The paired samples *T*-test indicated there was a significant difference between mean EOG and head onset in the pre-trials, but non-significant differences were found for cued and post trials. Data presented in Figure 2, suggest the cued trials slightly delayed NYA anticipatory eye movement. This slight delay in anticipatory eye movement did recover in the post trials once the cues were removed. These data suggest the appearance of the second discrete external visual cue was either later than normal for the neurotypical young adults, who in pre-trials began eye movement ~400 ms prior to IFC1, and/or that the instructions given to follow the numbers altered normal eye control in NYA. Therefore, instructions and timing of visual cues are a critical consideration when studying endogenous visual cues. Small differences in these factors could limit comparisons between studies.

### PD Participants

Individuals with PD are known to fixate on objects while turning which impedes anticipatory eye movements ahead of the turn, resulting in altered segment coordination during turning (Ambati et al., 2016; Stuart et al., 2017). This was evident, in the pre-trials of our study where anticipatory eye movements were not observed (Figure 1), and the resulting sequence of body reorientation occurred from the pelvis up (Figure 4). During cued trials some changes in sequences were observed though not statistically significant. However, it was on the removal of the discrete external visual cues for the post trials that a classic anticipatory eye and head first turning strategy was observed in PD (Figure 4). This was further supported by the differences between eye-head and head-trunk rotation onsets. This was a very interesting result and was the major contribution of this preliminary study to future work. It could be that visual cues do in fact train eye movements successfully and it would be interesting to see with longer post intervals how long PD retain anticipatory eye behavior following training.

Several limitations should be addressed moving forward from this preliminary report. The most notable was the low sample size of the PD group (*n* = 6). Secondly, only 90 degree left turns were studied. The decision to limit the turns to one side was made for two reasons: (1) to limit fatigue in PD (including left and right would double the trials and increase lab time); (2) previous research in turning has collapsed left and right turns because of non-significant differences. Gait speed was not controlled rather a self-selected pace was used. The decision to use a self-selected pace was made because auditory cues (e.g., a metronome) alter gait and turning control in PD (Spildooren et al., 2017), and the purpose of the current study was to examine visual cues. Despite not controlling gait speed, the time to turn was not significantly different between conditions. As this was a within group and within participant experimental design any differences across groups and participants in gait speed did not influence the use of time data. Analysis of EOG was very simple and greater analyses of these data could provide interesting research avenues.

Overall, this exploratory study is the first to examine the use of discrete external visual cues placed along a 90-degree walking turn with the goal of producing coordinated turning in individuals with PD. The results highlight that even the simplest design of discrete external visual cues can produce anticipatory eye movement and a head first turning strategy in PD. However, the positive effects of using visual cues with walking turns seems to be most evident *post* training, once visual cues are removed from the environment. These results have interesting implications for the use of visual cues during turning movements in studies aiming to improve turning control in populations who have difficulty. In addition, the results of this study further support the hypothesis that eye movement has a critical role in the motor control of turning movements.

## Data Availability Statement

The datasets generated for this study are available on request to the corresponding author.

## Ethics Statement

The studies involving human participants were reviewed and approved by University of Prince Edward Island Research Ethics Board. Written informed consent was obtained from the individual for the publication of any potentially identifiable images included in this article.

## Author Contributions

RR-J, TB, and JP contributed substantially to the conception and design of the work. RR-J, TB, JP, and MM contributed substantially to the analysis and interpretation of data for the work. All authors contributed to the drafting of the work or revising it critically for important intellectual content and provided approval for publication of the content and agree to be accountable for all aspects of the work in ensuring that questions related to the accuracy or integrity of any part of the work are appropriately investigated and resolved.

### Conflict of Interest

The authors declare that the research was conducted in the absence of any commercial or financial relationships that could be construed as a potential conflict of interest.

## Supplementary Material

Data Sheet 1 A schematic of the experimental layout. Each square represents 0.15 meters. Optotrak cameras are coded in blue and the four cues (0°, 40°, 60°, 90°) in yellow.
