# Supplementary figures and images for: Visual Cues Promote Head First Strategies During Walking Turns in Individuals With Parkinson's Disease
Source: Front Sports Act Living. 2020 Mar 11;2:22. doi: 10.3389/fspor.2020.00022 (PMC7739666; doi:10.3389/fspor.2020.00022)

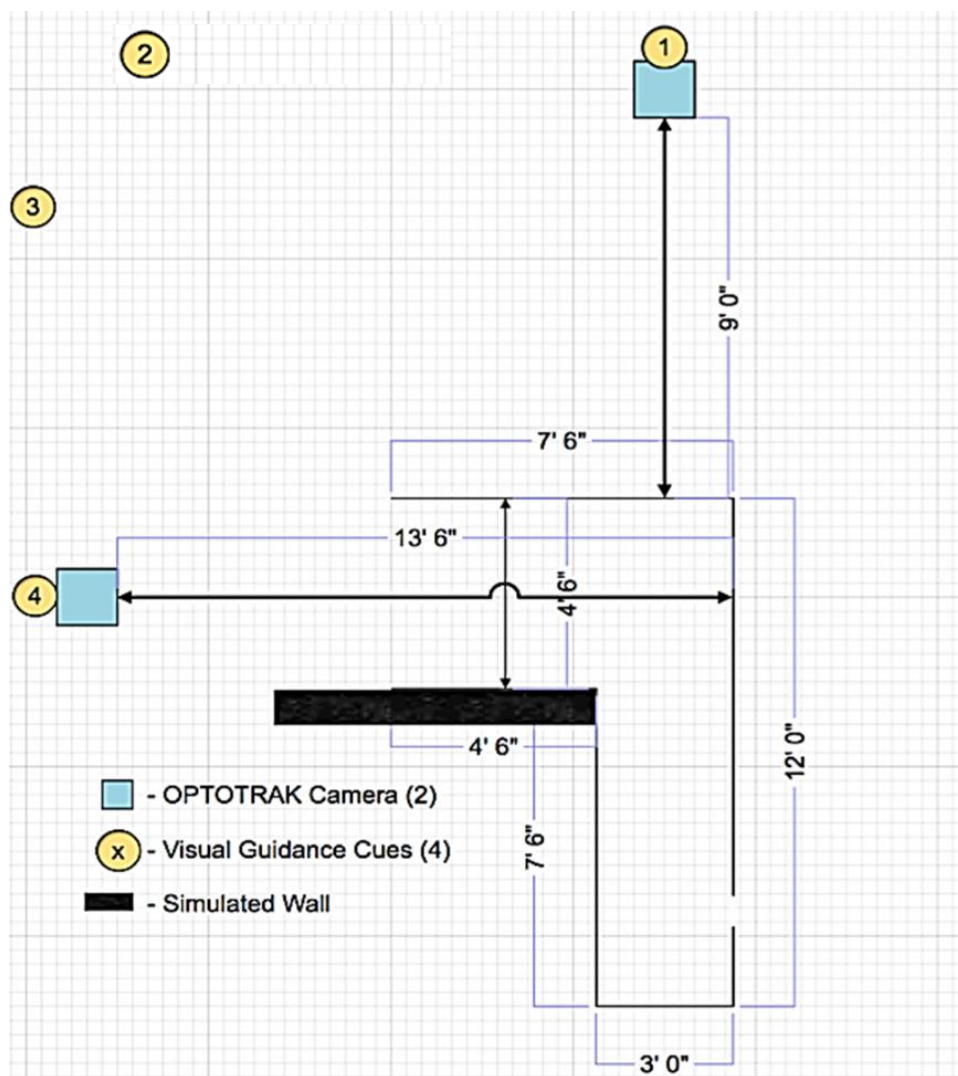

Supplement: Data Sheet 1 — A schematic of the experimental layout. Each square represents 0.15 meters. Optotrak cameras are coded in blue and the four cues (0°, 40°, 60°, 90°) in yellow. [file Data_Sheet_1.PDF]

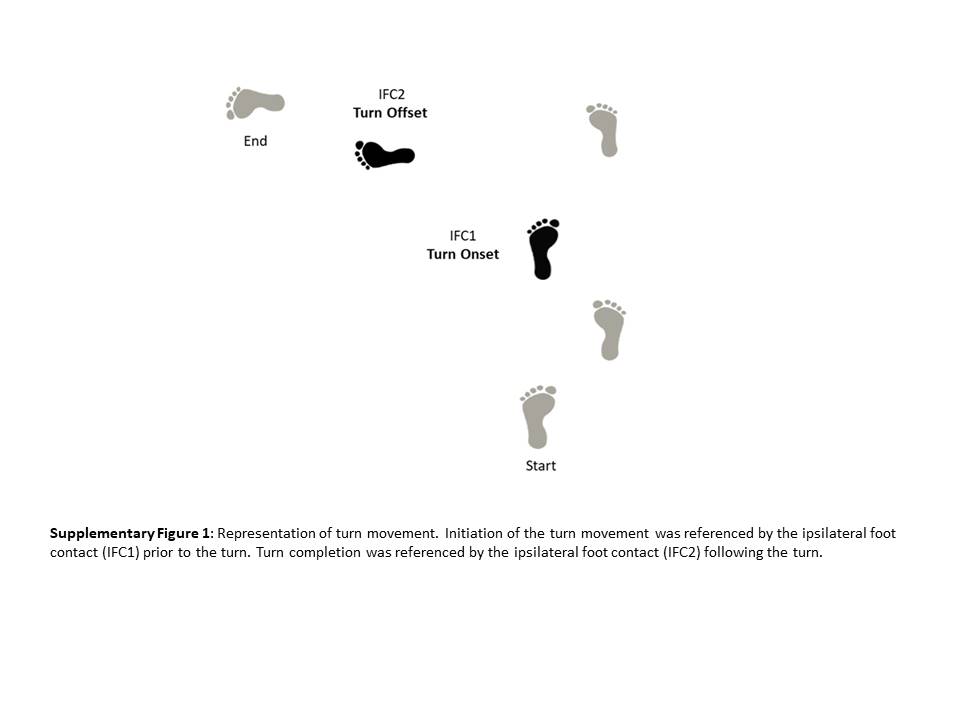

Supplement: Supplementary file 2 [file Image_1.JPEG]

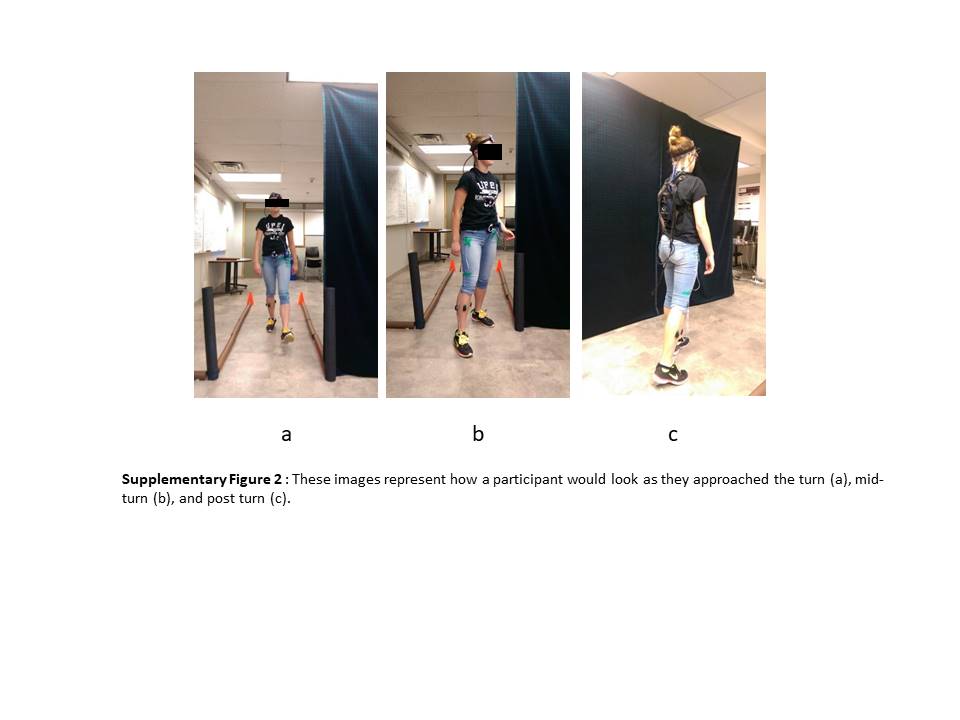

Supplement: Supplementary file 3 [file Image_2.JPEG]

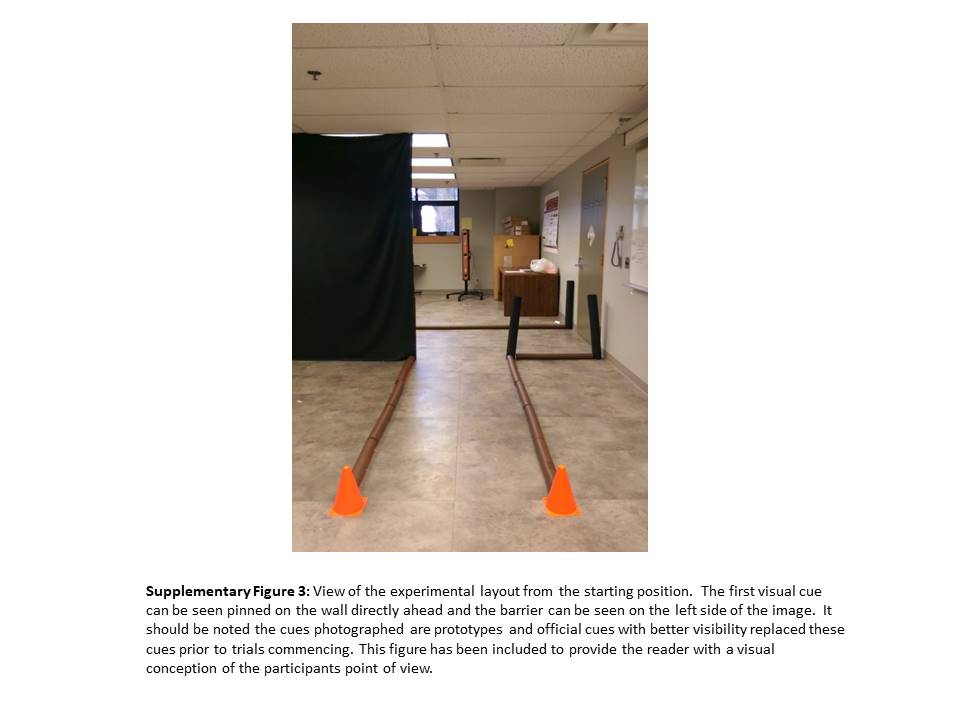

Supplement: Supplementary file 4 [file Image_3.JPEG]

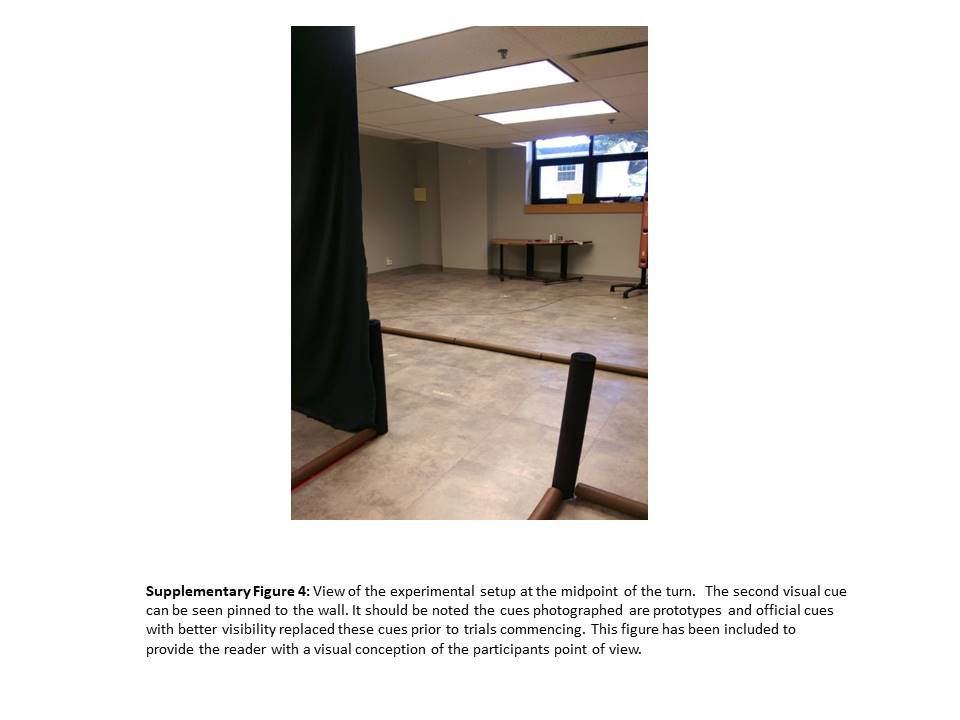

Supplement: Supplementary file 5 [file Image_4.JPEG]

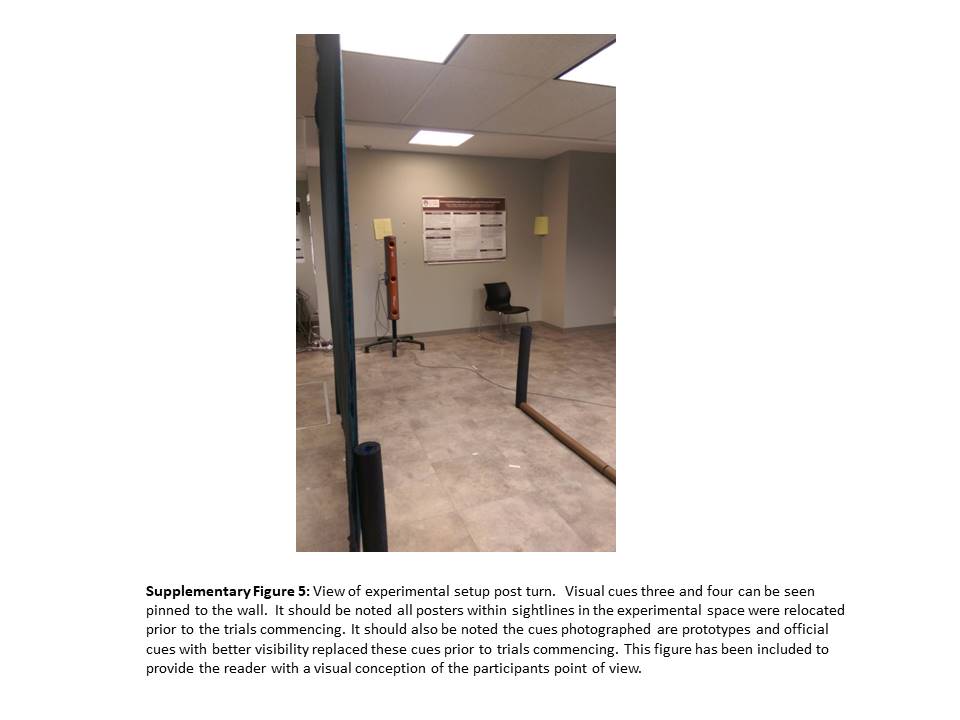

Supplement: Supplementary file 6 [file Image_5.JPEG]

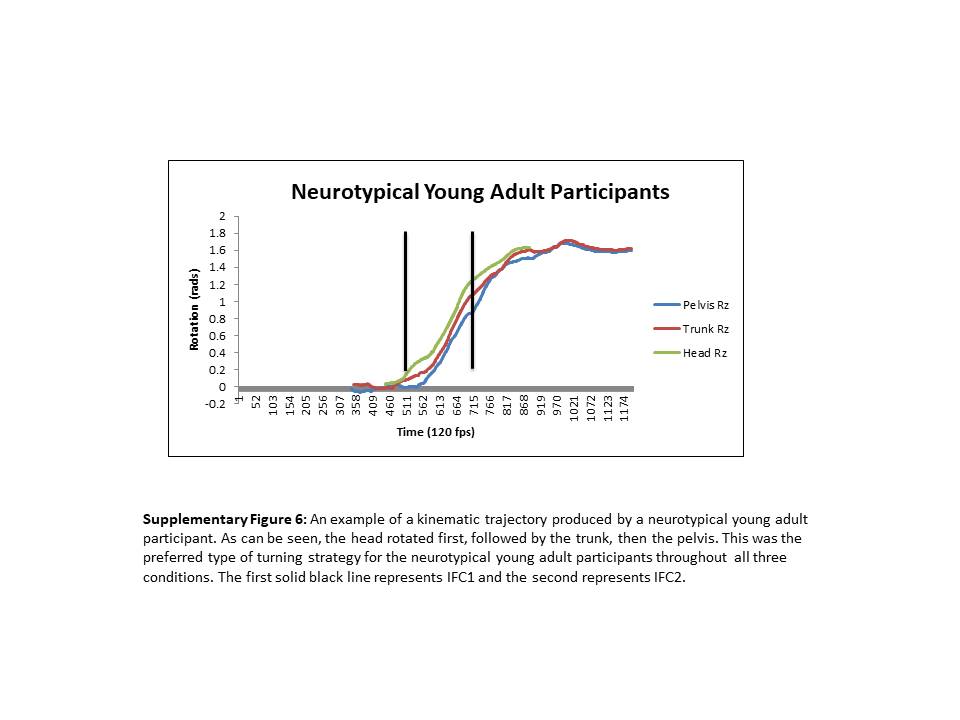

Supplement: Supplementary file 7 [file Image_6.JPEG]

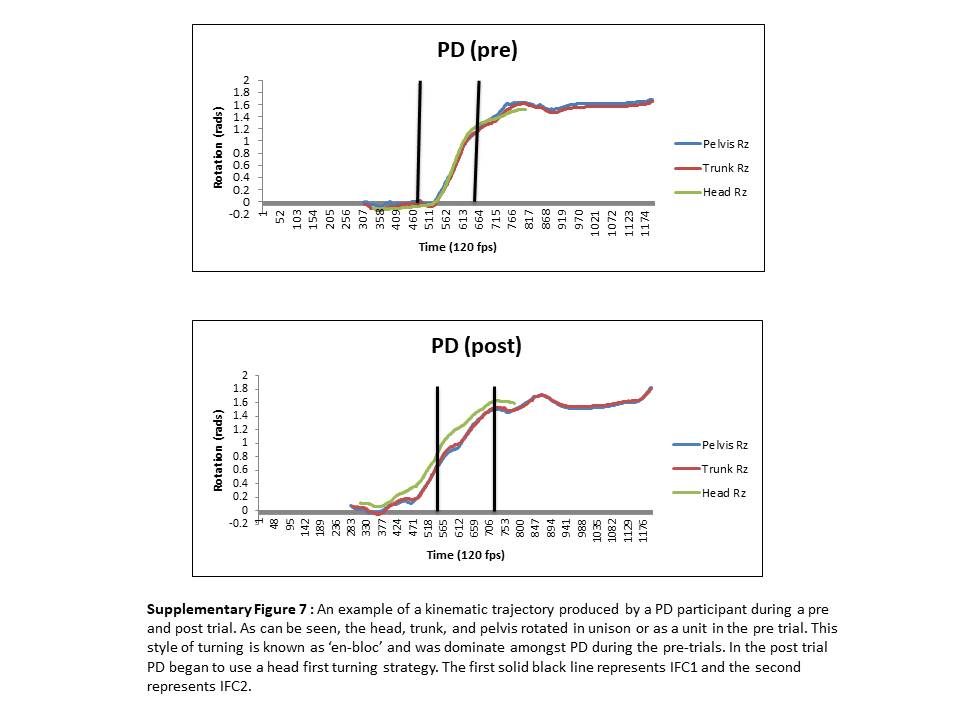

Supplement: Supplementary file 8 [file Image_7.JPEG]

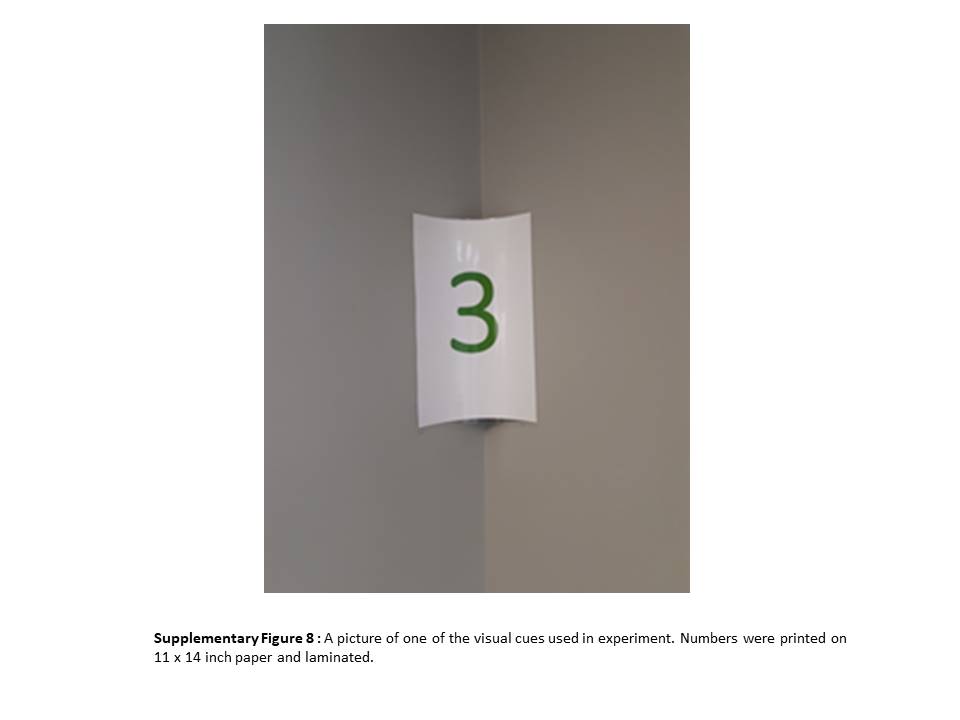

Supplement: Supplementary file 9 [file Image_8.JPEG]
